# The Small RNA AmiL Regulates Quorum Sensing-Mediated Virulence in Pseudomonas aeruginosa PAO1

**DOI:** 10.1128/spectrum.02211-21

**Published:** 2022-03-09

**Authors:** Jieying Pu, Shebin Zhang, Xi He, Jianming Zeng, Cong Shen, Yanfen Luo, Honglin Li, Yifei Long, Jianping Liu, Qian Xiao, Yang Lu, Bin Huang, Cha Chen

**Affiliations:** a Department of Laboratory Medicine, the Second Affiliated Hospital of Guangzhou University of Chinese Medicine, Guangzhou, China; b The Second Clinical College of Guangzhou University of Chinese Medicine, Guangzhou, China; c Department of Laboratory Medicine, the First Affiliated Hospital of Sun Yat-sen University, Guangzhou, China; Emory University School of Medicine

**Keywords:** *Pseudomonas aeruginosa*, small noncoding RNAs, AmiL, quorum sensing, virulence

## Abstract

Pseudomonas aeruginosa is an opportunistic and nosocomial pathogen of humans with hundreds of its virulence factors regulated by quorum sensing (QS) system. Small noncoding RNAs (sRNAs) are also key regulators of bacterial virulence. However, the QS regulatory sRNAs (Qrrs) that have been characterized in P. aeruginosa are still largely unknown. Here, sRNA AmiL (PA3366.1) in the *amiEBCRS* operon of PAO1 was identified as a novel Qrr by transcriptome sequencing (RNA-Seq). The expression of AmiL was negatively regulated by the *las* or *rhl* system, of which RhlR probably inhibited its transcription. AmiL deletion mutant and overexpressing strains were constructed in PAO1. Broad phenotypic changes were found, including reduced pyocyanin synthesis, elastase activity, biofilm formation, hemolytic activity, and cytotoxicity, as well as increased rhamnolipid production and swarming motility. AmiL appears to be a new regulator that influences diverse QS-mediated virulence. Furthermore, AmiL directly targeted PhzC, a key member of pyocyanin synthesis. AmiL also negatively regulated *lasI* expression in the early growth of PAO1, but predominantly increased *rhlI* expression and C4-HSL production in the middle and late stages. Therefore, a novel QS-sRNA signaling cascade of *las*/*rhl* (RhlR)-AmiL-PhzC/*las*/*rhl* was demonstrated, and it will help to shed new light on the virulence regulatory network of P. aeruginosa PAO1.

**IMPORTANCE** P. aeruginosa is a common nosocomial pathogen that causes diverse opportunistic infections in humans. The virulence crucial for infection is mainly regulated by QS. Small noncoding RNAs (sRNAs) involved in virulence regulation have also been identified in many bacteria. Recently, there is a growing interest in the new sRNA species, QS regulatory sRNAs (Qrrs). Understanding Qrrs-mediated regulation in P. aeruginosa virulence is therefore important to combat infection. In this study, a previously uncharacterized sRNA AmiL in PAO1 has been identified as a novel Qrr. It has been found to influence diverse QS-mediated virulence factors including pyocyanin, elastase, rhamnolipid, and hemolysin, as well as biofilm formation, swarming motility, and cytotoxicity. Furthermore, PhzC essential for pyocyanin synthesis was a direct target of AmiL. QS gene expression and C4-HSL production were also regulated by AmiL. This study provides insights into the roles of Qrr AmiL in modulating P. aeruginosa virulence.

## INTRODUCTION

Pseudomonas aeruginosa is a Gram-negative bacterium that causes diverse opportunistic infections in a wide category of patients with burns, traumatized cornea, open fractures, lung diseases, or other immunocompromised diseases (1). Among the reasons that P. aeruginosa has been such a formidable pathogen include its intrinsic ability to develop resistance to antibiotics, form biofilms, and release amounts of virulence factors. The pathogenicity of P. aeruginosa is commonly associated with its diverse virulence factors, mainly including pyocyanin, rhamnolipid, elastase, hemolysin, lipopolysaccharide, and other secreted molecules (2, 3). Virulence factors are beneficial for P. aeruginosa to enhance colonization, disrupt the host immune responses, and finally establish infection. In addition, biofilms in P. aeruginosa lead to many persistent and non-invasive human infections, such as cystic fibrosis (CF), chronic wound infection, and medical device-associated infections (4).

Quorum sensing (QS) is a cell density-based bacterial intercellular communication system that enables the individual cells to act as a community. Various QS systems in bacteria that regulate virulence gene expression and biofilm formation have been found. In P. aeruginosa, there are currently four known connected QS systems including *las*, *rhl*, *pqs*, and *iqs* (5), which controls the expression of more than 300 genes involved in virulence regulation (6). The *las* and *rhl* are *N*-acylated homoserine lactone (AHL) signaling systems. In *las* system, the autoinducer synthase LasI produces 3-oxo-C12-HSL, which interacts with LasR receptor (also known as a transcription factor) to activate certain virulence factors, mainly including LasB elastases, LasA protease, exotoxin A, and alkaline protease (7–9). Similarly, the autoinducer synthase RhlI in *rhl* system produces C4-HSL, which interacts with RhlR receptor to produce other virulence factors, such as rhamnolipid, chitinase, and hydrogen cyanide (10, 11). RhlR is also known as a transcription factor of the *rhl* system. The third system, *pqs*, its autoinducer synthase PqsABCD produces quinolones (PQS signals), which binds PqsR receptor to regulate pyocyanin production (12). The final system is *iqs*, which has AmbBCDE autoinducer synthase to produce IQS signals (13). The four QS systems in P. aeruginosa are organized hierarchically, with *las* is often described as being at the top. LasR/3-oxo-C12-HSL network positively regulates the gene expression of RhlI, RhlR, and PqsR (14, 15). The *pqs* system also regulates *las* and *rhl* through its PQS signals (16). The QS regulatory network was well-coordinated, allowing P. aeruginosa to fine-tune its response to cell population changes, and govern virulence genes expression.

Bacterial small noncoding RNAs (sRNAs) are important RNA species characterized in prokaryotes. sRNAs are critical players in the post-transcriptional regulation of various genes in a positive- or negative manner, by base-pairing with their target mRNAs (17). Generally, sRNAs appear to adjust bacterial physiology in response to environmental changes or stress conditions. In recent years, increasing studies have shown that sRNAs are involved in bacterial pathogenesis, including QS and virulence regulation (18–20). Using transcriptome sequencing (RNA-Seq), hundreds of potential intergenic sRNAs were identified in P. aeruginosa (21, 22). Nevertheless, few of these novel sRNAs have been functionally characterized, especially their roles in QS regulation. The P. aeruginosa sRNA ReaL, negatively regulated by LasR, acts as a link between the *las* and *pqs* systems by targeting pqsC and positively regulating its translation, which finally results in increased pyocyanin production and biofilm formation (23). Subsequently, ReaL is also shown to base-pair with rpoS mRNA to negatively control RpoS synthesis, which is involved in the regulation of QS (24). A previous study in our lab found sRNA PrrH directly represses LasI and its expression is negatively regulated by the *rhl* system, and thus involves in the regulation of virulence factors, biofilm, and motility (25). In addition, sRNA PhrS and PrrF modulate the *pqs* system by regulating PqsR and AntR, respectively (26, 27). However, the above QS regulatory sRNAs (Qrrs) that have been characterized in P. aeruginosa are limited. Further studies of novel Qrrs will help to disclose the virulence regulatory network of P. aeruginosa.

To investigate novel Qrrs of P. aeruginosa, a QS activating model, *lasI* gene-deficient strain, and *rhlI* gene-deficient strain in PAO1 were constructed. Under these conditions, the transcription of sRNA species was identified by RNA-Seq, a technology that has been widely used to screen QS-controlled genes in P. aeruginosa (28–30). One of those screening sRNAs, a novel one, AmiL (PA3366.1), was negatively regulated by QS, and its expression changes were confirmed by quantitative reverse transcriptase PCR (qRT-PCR). The 100 nt sRNA AmiL is a leader of the *amiEBCRS* operon of PAO1 (31, 32). In the *amiEBCRS* operon, *amiL* gene precedes *amiE*, a gene that encodes aliphatic amidase AmiE (33, 34). AmiE is a well-studied enzyme that involves in carbon-nitrogen metabolic processes (35, 36). Recent research found AmiE could exercise additional functions in regulating P. aeruginosa virulence (37). However, the role of AmiL has rarely been studied, even though its gene is located on the same operon as *amiE*. AmiL was thus considered for further analysis in this study. Phenotypic characterizations of *amiL* gene-deficient or overexpressing strains showed significant roles for AmiL in regulating pyocyanin, elastase, rhamnolipid, biofilm, swarming motility, hemolysin, and mammalian cytotoxicity. Additional studies found AmiL directly targeted PhzC, a key member from the *phz* operon for pyocyanin synthesis. AmiL also negatively regulated *lasI* expression in the early growth of PAO1, but predominantly increased *rhlI* expression and C4-HSL production in the middle and late stages. Therefore, the sRNA AmiL is a novel Qrr that influences diverse QS-mediated virulence in P. aeruginosa PAO1.

## RESULTS

### Identification of a novel QS regulatory sRNA AmiL by RNA-Seq.

**FIG 1:**
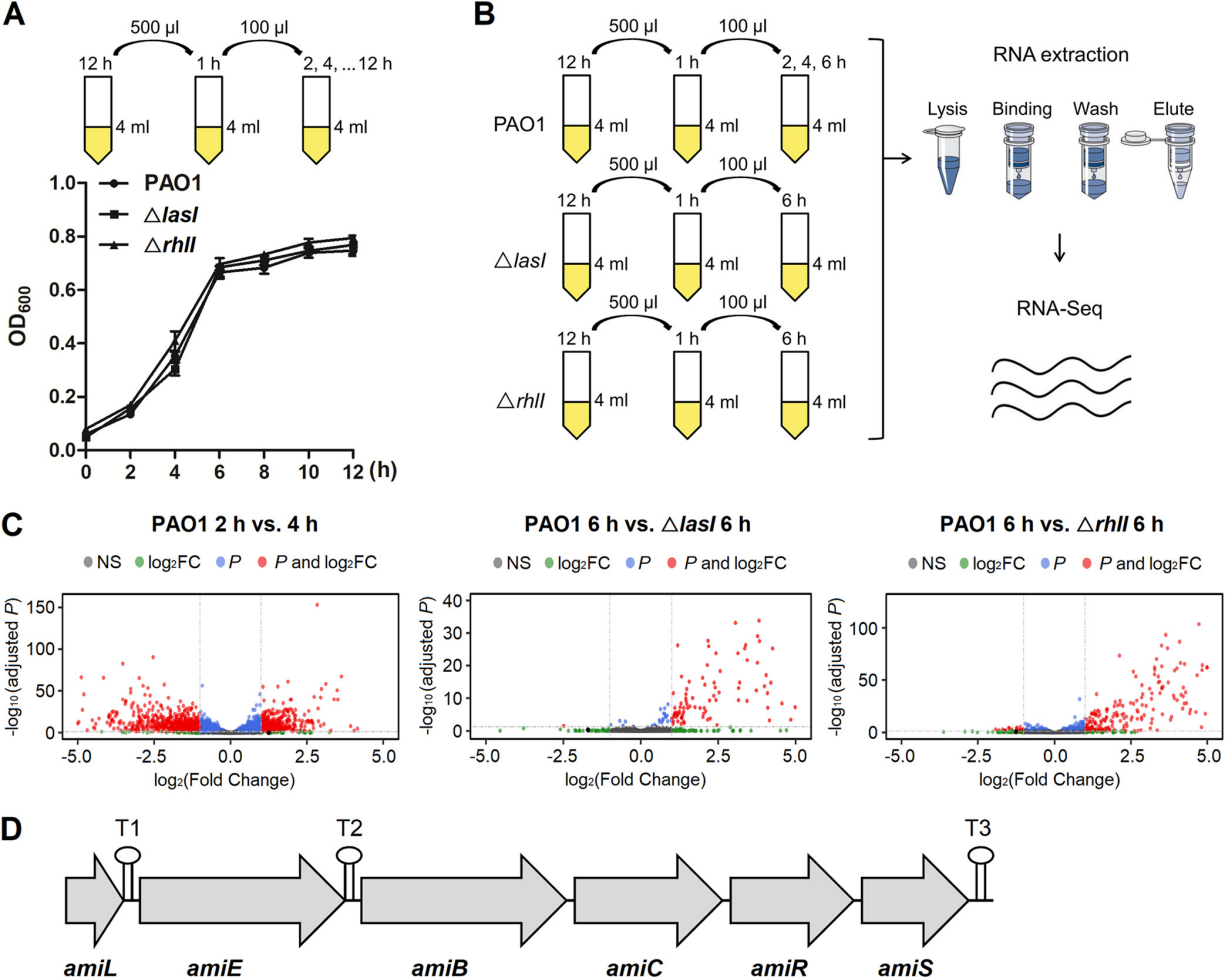
AmiL as a novel QS regulatory sRNA was identified by RNA-Seq. (A) The QS activation model, and growth curves of PAO1 and its *lasI* or *rhlI* gene-deficient strain (△*lasI*, △*rhlI*). (B) RNA-Seq study of PAO1 which was under QS activation (PAO1, *t* = 2 h, 4 h, 6 h) or QS inhibition (△*lasI*, △*rhlI*, *t* = 6 h) condition. (C) Volcano plot analysis of RNA-Seq results, the groups that PAO1 2 h versus 4 h and PAO1 6 h versus △*lasI* 6 h or △*rhlI* 6 h were showed with over 6,000 variables; AmiL, the black dot. (D) Genetic organization of the *amiEBCRS* operon, and transcription termination sequences are shown as a stem-loop symbol (T1, T2, T3).

To investigate novel Qrrs in P. aeruginosa, we first constructed a QS activating model of PAO1. Given that the activation of QS depends on bacteria density, PAO1 and its *lasI* or *rhlI* gene-deficient strain (△*lasI*, △*rhlI*) were cultured respectively overnight and inoculated in fresh Luria-Bertani (LB) twice to have a rapid enrichment (Fig. 1A, top). Bacterial growth curves were showed no difference, including the early exponential phase (*t* = 2 h), the mid-exponential phase (*t* = 4 h), and the early stationary phase (*t* = 6 h) (Fig. 1A, bottom). We further performed RNA-Seq under QS activation (*t* = 2 h, 4 h, 6 h) and QS genes deletion (*t* = 6 h) conditions to screen the Qrrs in PAO1 (Fig. 1B). In brief, a single clone from PAO1, △*lasI*, or △*rhlI* was grown in fresh LB medium overnight, then the culture from the second grow of 1 h was incubated for indicated time before sequencing. The volcano plots of PAO1 2 h versus 4 h and PAO1 6 h versus △*lasI* 6 h or △*rhlI* 6 h were shown (Fig. 1C). RNA-Seq result revealed that 15 sRNA species were dysregulated under the QS activation or inhibition condition (Table S1). *P* value and absolute log_2_ fold change (logFC) were also analyzed (Table S2, S3). We found that these sRNA species are part of 29 annotated sRNAs of PAO1 in the NCBI website (https://www.ncbi.nlm.nih.gov/gene/?term=ncRNA+and+PAO1). Significantly, the expression of sRNA AmiL (PA3366.1) was decreased in the mid-exponential phase of PAO1 growth, but it was upregulated in △*lasI* and △*rhlI* strains. AmiL expression changes in QS activation and inhibition conditions showed high consistency. AmiL is a leader of the *amiEBCRS* operon in PAO1, thus we showed the genetic organization of this operon (Fig. 1D), and also analyzed the RNA-Seq data about the expression of the genes (Table S4). Therefore, AmiL was identified as a novel QS regulatory sRNA that was chosen for further analysis in detail by virulence phenotypic screens.

### Negative regulation of AmiL transcription by RhlR.

**FIG 2:**
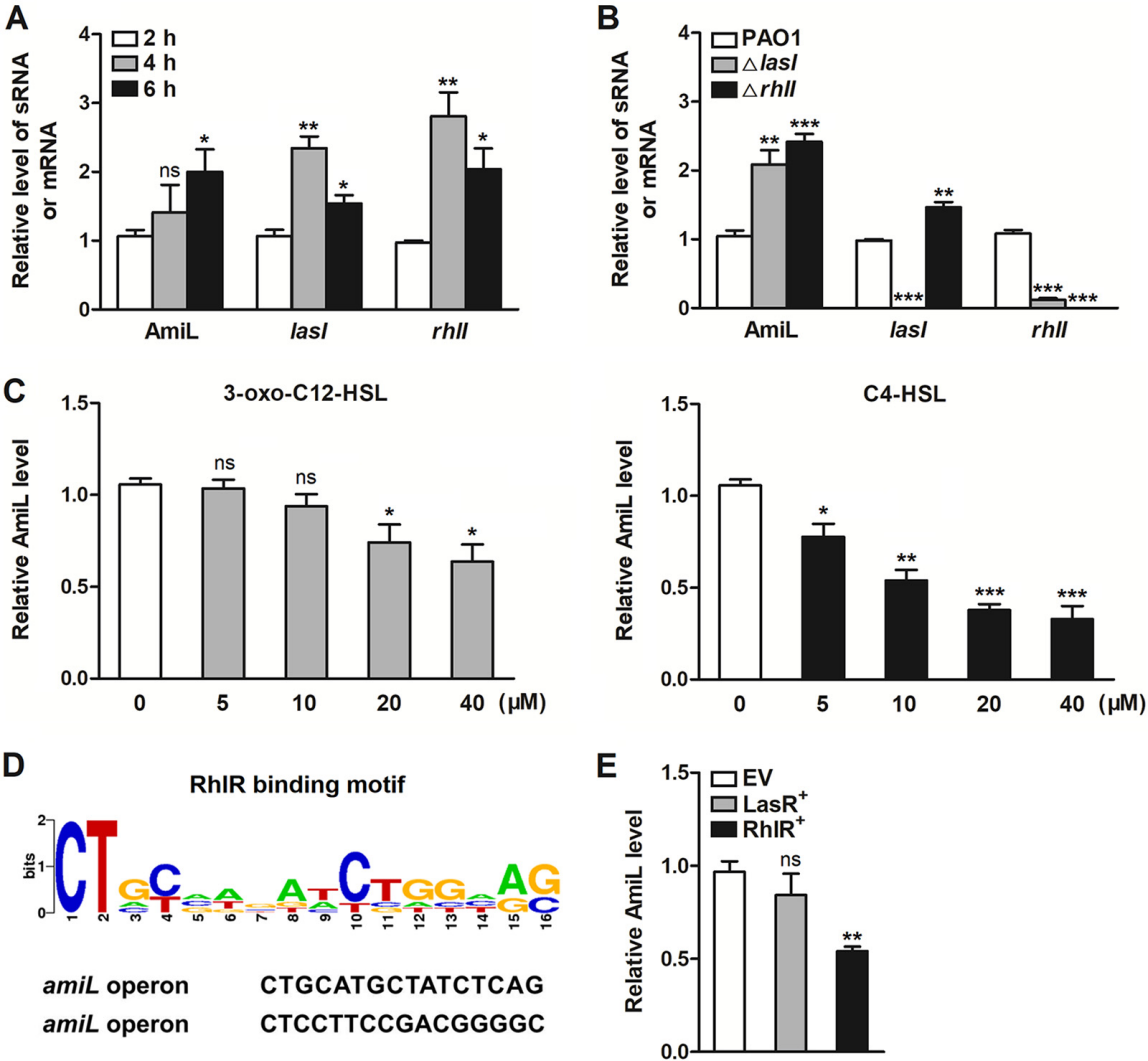
The transcription of AmiL was probably inhibited by RhlR. (A, B) AmiL expression levels in QS activation and inhibition conditions were detected by qRT-PCR. The activation model of QS (*t* = 2 h, 4 h, 6 h) was used to analyze the expression of AmiL, *lasI*, and *rhlI* (A). At 6 h of growth, the expression levels of AmiL, *lasI*, *rhlI* in PAO1, and *lasI* or *rhlI* gene-deficient strain (△*lasI*, △*rhlI*) were shown, respectively (B). (C) After treatments with 3-oxo-C12-HSL or C4-HSL with concentrations ranging from 5 to 40 μM for 6 h, AmiL expression levels in PAO1 were measured by qRT-PCR, compared with the vehicle DMSO (0 μM). (D) Diagram of RhlR-binding motif in the operon of *amiL*, the putative target gene. (E) PAO1 carrying the pROp200 empty vector (EV), pROp200-*lasR* (LasR^+^), or pROp200-*rhlR* (RhlR^+^) was cultured in LB for 6 h, and AmiL expression was measured by qRT-PCR. Data are shown as mean ± SEM of at least three independent experiments. *, *P < *0.05; **, *P < *0.01; ***, *P < *0.001; ns, non-significant.

Screening by RNA-Seq, AmiL was identified as a novel QS regulatory sRNA. We first confirmed the expression levels of AmiL in QS activation and inhibition conditions by qRT-PCR. The culture conditions of PAO1, strain △*lasI*, or △*rhlI* were the same as described in Fig. 1B. Surprisingly, AmiL level showed no decrease in the mid-exponential phase (*t* = 4 h) of PAO1 growth and had a little increase in the early stationary phase (*t* = 6 h) (Fig. 2A). However, the expression of AmiL in △*lasI* or △*rhlI* strain was upregulated obviously (*t* = 6 h), which was consistent with RNA-Seq (Fig. 2B). To further confirm the influence of *las* and *rhl* systems on AmiL expression, treatment with QS signal molecules with concentrations ranging from 5 to 40 μM was employed. As shown in Fig. 2C, there was little inhibition in AmiL expression only when 3-oxo-C12-HSL concentration reached 20 μM or 40 μM. However, all concentration treatments with C4-HSL significantly inhibited the expression of AmiL in a dose-dependent manner. LasR and RhlR are the transcription factors of *las* and *rhl*, respectively. To investigate whether AmiL transcription was regulated by LasR or RhlR, the bioinformatics tool PRODORIC, a comprehensive database for gene regulation and expression in prokaryotes, was used to analyze the DNA motif recognized by LasR or RhlR. It showed that there were two putative RhlR binding sites in the −150 bp region upstream of AmiL, with the specific DNA sequence 5′-CT(N12)AG-3′ or 5′-CT(N12)GC-3′ in this region (Fig. 2D). Next, we constructed LasR or RhlR overexpressing strain in PAO1, termed LasR^+^ and RhlR^+^. The strains of empty vector (EV), LasR^+^, and RhlR^+^ were cultured in fresh LB medium for 6 h. Consistent with bioinformatics data, overexpression of RhlR significantly decreased AmiL expression, but LasR did not (Fig. 2E). These results suggest RhlR may be involved in the regulation of AmiL transcription.

### AmiL involvement in the regulation of different virulence phenotypes.

**FIG 3:**
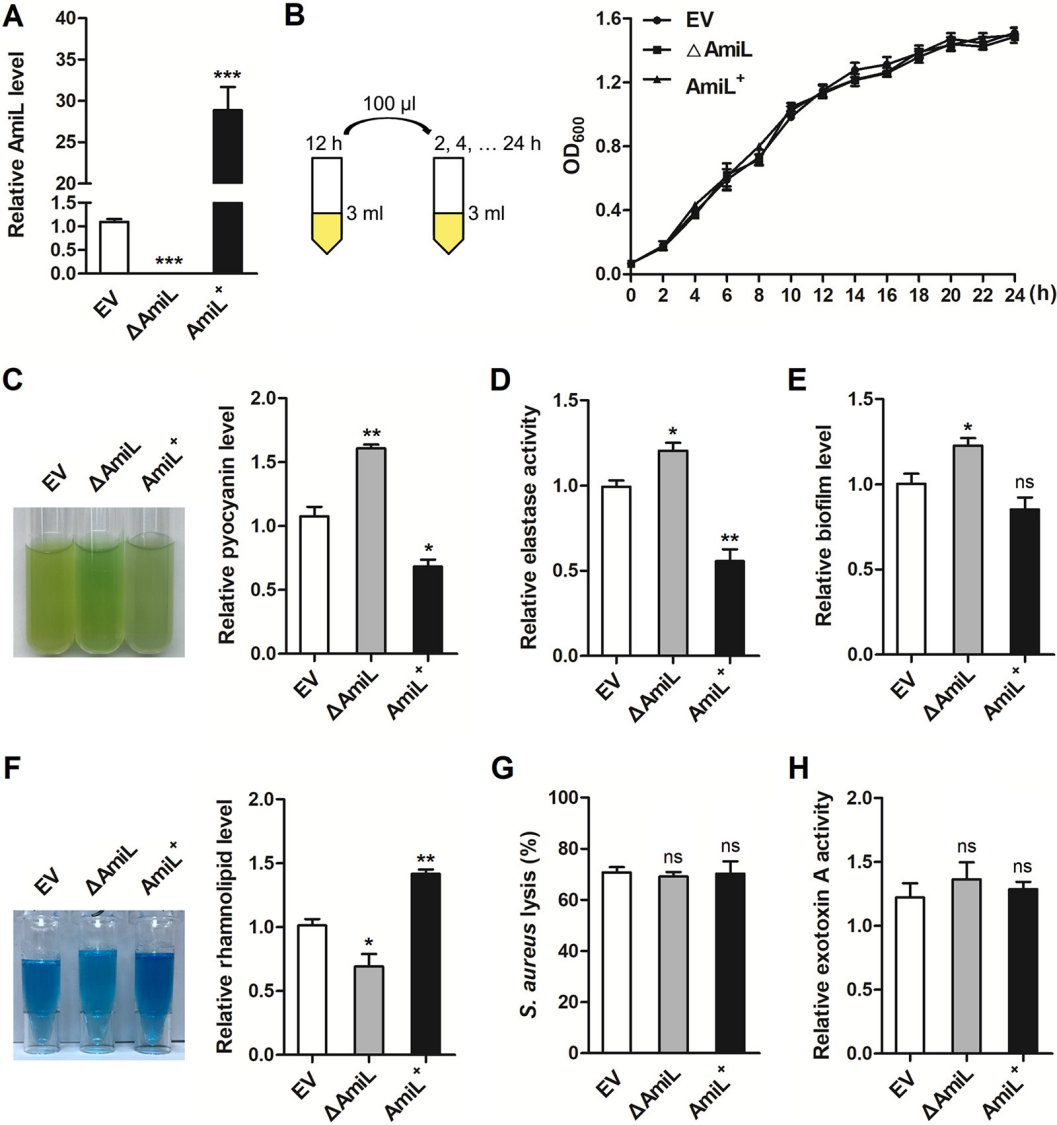
AmiL influenced pyocyanin and rhamnolipid production, elastase activity, and biofilm formation of P. aeruginosa. (A) PAO1 carrying the pROp200 empty vector (EV) or pROp200-*amiL* (AmiL^+^), and *amiL*-deleted PAO1 carrying pROp200 (△AmiL) were cultured in LB for 6 h, and AmiL expression was measured by qRT-PCR. (B) Growth curve analysis of the EV, △AmiL, and AmiL^+^ strains. (C) Pyocyanin in the supernatant of EV, △AmiL, and AmiL^+^ strains that cultured in LB for 24 h was detected. (D) The activity of elastase in the supernatant of EV, △AmiL, and AmiL^+^ strains cultured in LB for 8 h was measured. (E) Biofilm formation of the indicated strains that cultured in LB in the 96-well plate for 24 h was determined. (F) The indicated AmiL-constructed strains were cultured in M9 medium for 16 h, and rhamnolipid in the supernatant was measured. (G) The EV, △AmiL, and AmiL^+^ strains were cultured in LB for 24 h, and Staphylococcus aureus was used to perform the LasA staphylolytic assays. (H) Exotoxin A activity of the indicated strains cultured in Trypticase soy broth (TSB) with 1% glycerol for 24 h was measured. Data are shown as mean ± SEM of at least three independent experiments. *, *P < *0.05; **, *P < *0.01; ***, *P < *0.001; ns, non-significant.

QS system plays a key role in the regulation of bacterial virulence. As a QS regulatory sRNA, AmiL probably influences P. aeruginosa virulence. To investigate this further, we constructed *amiL* gene-deficient (△AmiL) and overexpressing AmiL^+^ strains in PAO1. The strains of EV, △AmiL, and AmiL^+^ were cultured in fresh LB medium for 6 h, and AmiL expression level was confirmed by qRT-PCR. AmiL level in the AmiL^+^ strain was averagely upregulated 28.9-fold than that in the EV stain, and its expression in the △AmiL strain was undetected (Fig. 3A). Bacterial growth curves of AmiL strains were also measured. The result in Fig. 3B showed that AmiL deletion or overexpression did not affect the growth of PAO1. We first investigated the role of AmiL in regulating pyocyanin synthesis. A single clone from EV, △AmiL, or AmiL^+^ was cultured in LB medium overnight and then had a second grow for 24 h. Compared with the EV strain, AmiL deletion mutant had increased production of pyocyanin significantly, while AmiL overexpressing strain resulted in a reduction (Fig. 3C). The elastase activity, biofilm formation, and rhamnolipid production were subsequently measured. The indicated EV, △AmiL, and AmiL^+^ strains from an overnight culture were under a second grow for 8 h (elastase), 24 h (biofilm), and 16 h (rhamnolipid), respectively, before the assays. Of which, elastase activity was determined by elastin-Congo red (ECR) assays, biofilm detection was performed with crystal violet staining in a 96-well plate, and rhamnolipid was measured by methylene blue complexation. As shown in Fig. 3D and E, deletion of AmiL found increases of elastase activity and biofilm formation in PAO1. AmiL overexpression similarly reduced elastase activity but showed no or little inhibition of biofilm formation. It seems that the regulation of AmiL on biofilm formation is not as strong as other virulence phenotypes. Different from the results of pyocyanin, elastase, and biofilm, rhamnolipid production was downregulated in AmiL deletion mutant and was enhanced by AmiL overexpression (Fig. 3F). We finally investigated the impact of AmiL on staphylolytic (Staphylococcus aureus lysis) activity and exotoxin A activity of PAO1, but found no statistical significance (Fig. 3G and H).

**FIG 4:**
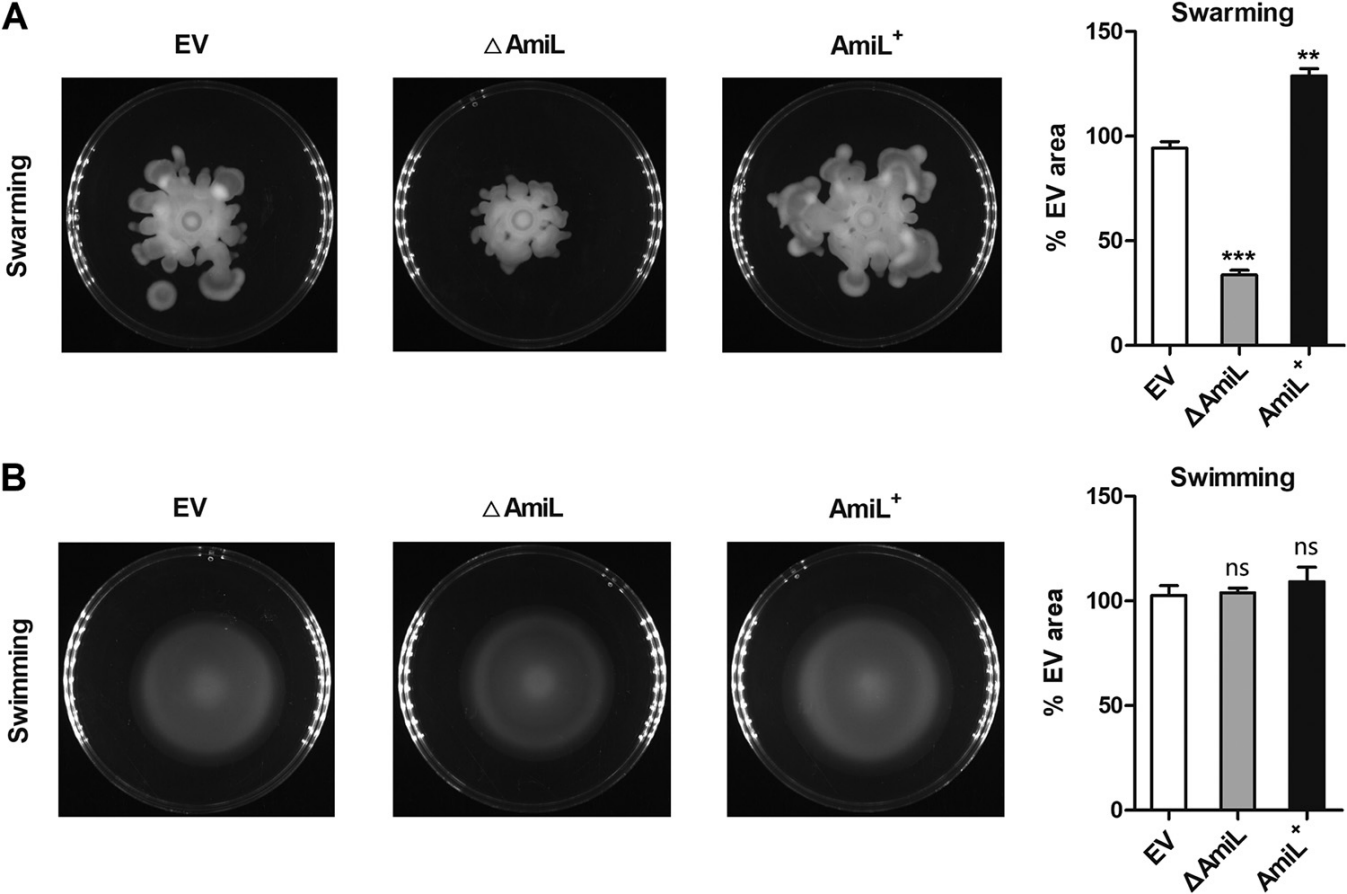
AmiL positively regulated swarming motility, but had no impact on swimming. The strains, PAO1 carrying the pROp200 empty vector (EV) or pROp200-*amiL* (AmiL^+^), and *amiL*-deleted PAO1 carrying pROp200 (△AmiL) were used. (A) 5-μL cultures of the indicated strains were spotted onto the swarming medium and incubated for 16 h; swarming motility assays were performed. (B) 5-μL cultures of the indicated strains were spotted onto the swimming medium and incubated for 16 h; swimming motility assays were adopted. Data are shown as mean ± SEM of at least three independent experiments. **, *P < *0.01; ***, *P < *0.001; ns, non-significant.

Bacterial motility is another QS-mediated virulence phenotype that involves both pili and flagella to enhance infection for P. aeruginosa. We next analyzed the roles of AmiL in swarming and swimming motilities of PAO1. After 16 h-culture in swarming or swimming medium, the motility assays showed that AmiL deletion resulted in an obvious reduction in swarming (averagely reduced to 33.6% of EV), and AmiL overexpression averagely increased to 128.8% of EV (Fig. 4A). However, there was no regulation of swimming in *amiL*-deficient or overexpressing strain that was compared with the EV (Fig. 4B). Therefore, these results from virulence phenotypic screens indicated that AmiL plays negative roles in regulating pyocyanin synthesis, elastase activity, and biofilm formation, while its roles in rhamnolipid production and swarming motility are positive.

### Effects of AmiL on hemolytic activity and mammalian cytotoxicity of P. aeruginosa.

**FIG 5:**
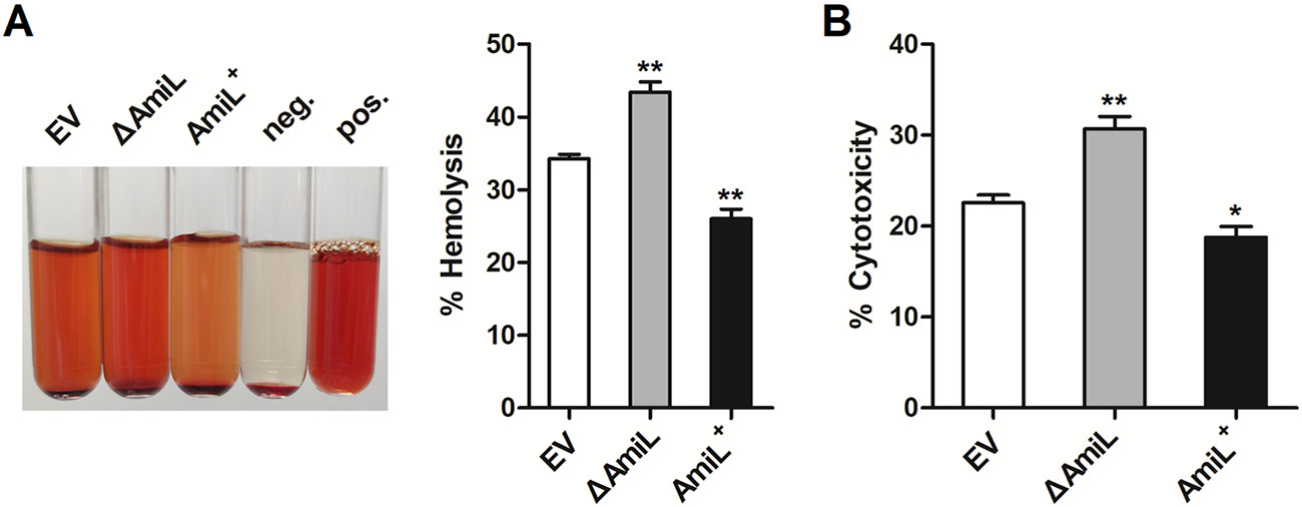
AmiL involved in regulating hemolytic activity and mammalian cytotoxicity of P. aeruginosa. (A) PAO1 carrying the pROp200 empty vector (EV) or pROp200-*amiL* (AmiL^+^), and *amiL*-deleted PAO1 carrying pROp200 (△AmiL) were washed with PBS, and then 4% human erythrocytes were treated respectively with the indicated PBS-washed strains, human blood hemolysis assays were performed (2% Triton X-100 was used as a positive control, PBS as a negative control). (B) The indicated strains were well cocultured with A549 cells (MOI of 4) for 12 h, and then cytotoxicity assays by monitoring the release of lactate dehydrogenase (LDH) as an indicator was performed. Data are shown as mean ± SEM of at least three independent experiments. *, *P < *0.05; **, *P < *0.01.

Hemolysin is one of the important virulence factors of P. aeruginosa regulated by QS. The role of AmiL in hemolysis activity of PAO1 was thus investigated by human blood hemolysis assays. EV, △AmiL, and AmiL^+^ strains were cocultured with washed erythrocytes for 4 h. As shown in Fig. 5A, AmiL deletion significantly upregulated the hemolysis rate of PAO1, and overexpression of AmiL decreased it. Subsequently, the AmiL deletion mutant and overexpressing strains were also tested for cytotoxicity against human pulmonary epithelial cells (A549). The coculture of indicated strain with A549 was incubated for 12 h, followed by monitoring the release of lactate dehydrogenase (LDH) as an indicator of cytotoxicity. Likewise, AmiL deletion mutant resulted in stronger cytotoxicity against A549 cells, and its overexpressing strain reduced the cytotoxicity (Fig. 5B). Thus, these data revealed that AmiL has decreased hemolytic activity and cytotoxicity against mammalian cells.

### Involvement of AmiL in regulating PhzC, and the *las* and *rhl* systems.

**FIG 6:**
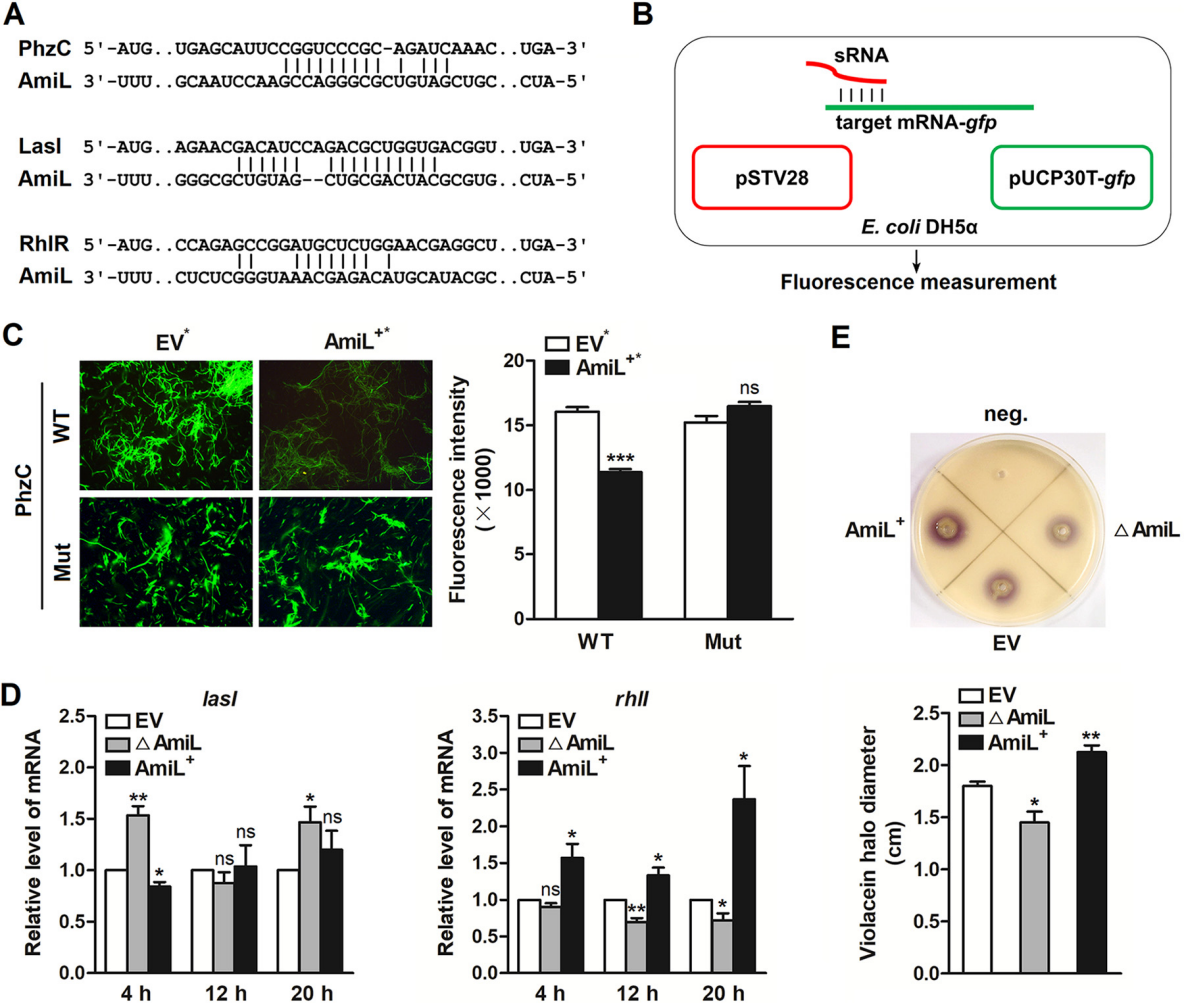
AmiL directly targeted PhzC, and showed significant regulations of the *las* and *rhl* systems. (A) AmiL and its putative binding sequence in the CDS of PhzC, LasI, and RhlR. (B) A green fluorescent protein (GFP) reporter system was constructed to investigate the direct interactions between sRNA and its potential targets. (C) The plasmid pSTV28 (EV*) or pSTV28-*amiL* (AmiL^+^*) was co-transformed with a GFP reporter plasmid (pUCP30T-*gfp*) containing a wild-type (WT) or mutant (Mut) sequences of PhzC mRNA into E. coli DH5α, then the fluorescence was measured by microscopy, and the intensity was detected by a BioTek Synergy H1 microplate reader and expressed in AU as F485/535. (D) PAO1 carrying the pROp200 EV or pROp200-*amiL* (AmiL^+^), and *amiL*-deleted PAO1 carrying pROp200 (△AmiL) were cultured in LB for 4 h, 12 h, and 20 h, respectively, then the expression levels of *lasI* (left) and *rhlI* (right) were measured by qRT-PCR. (E) The EV, △AmiL, and AmiL^+^ strains were added into the well of LB agar plates containing C. violaceum CV026 and incubated at 30°C for 24 h, the violacein halo production was observed (top) and the diameter of the halo was measured (bottom). Data are shown as mean ± SEM of at least three independent experiments. *, *P < *0.05; **, *P < *0.01; ***, *P < *0.001; ns, non-significant.

To explore the molecular mechanisms further, we firstly used the publicly available database IntaRNA to predict the target genes of AmiL. PhzC, LasI, and RhlR were potential targets (Fig. 6A). They are associated with P. aeruginosa virulence, of which PhzC plays a vital role in pyocyanin synthesis, while LasI and RhlR are the key members of QS. To further confirm the predicted data of IntaRNA, a green fluorescent protein (GFP) reporter system was developed (Fig. 6B). The predicted sequences (mRNA) of AmiL-targeted genes were inserted upstream of the *gfp* gene to construct a pUCP30T-mRNA-*gfp* plasmid. Then the plasmid was co-transformed into E. coli DH5α with AmiL overexpressing plasmid (AmiL^+^*) which was generated from pSTV28 (EV*), and the fluorescence intensity was measured by a microplate reader and fluorescence microscope. As shown in Fig. 6C, overexpression of AmiL significantly decreased the intensity of GFP that carried a wild-type (WT) but not mutant (Mut) base-pairing site of PhzC. However, it did not influence LasI or RhlR (data not shown). These results indicate that neither the predicted LasI nor RhlR are direct targets of AmiL. Next, the role of AmiL in the expression of QS genes (*lasI* and *rhlI*) was measured. The indicated EV, △AmiL, and AmiL^+^ strains were cultured in fresh LB medium for 4 h, 12 h, and 20 h, respectively, then the expression levels of *lasI* and *rhlI* were detected by qRT-PCR. For *lasI*, an obvious upregulation was found in AmiL deletion mutant after 4 h of growth, and a decrease was also observed in AmiL overexpressing strain (Fig. 6D, left). However, *lasI* expression was hardly affected by AmiL in 12 h or 20 h culture. For *rhlI*, deletion of AmiL significantly downregulated its expression after 12 h and 20 h of growth, consistently with the results from AmiL overexpressing strain (Fig. 6D, right). In the 4 h culture group, the expression of *rhlI* showed no or just a little change. The results from Fig. 6D suggest that AmiL may have a stronger influence on the *rhl* system. To get further insights, the AHLs biosensor Chromobacterium violaceum Tn5-mutant CV026, which mainly detects short-chains AHLs (38–40), was used to investigate the role of AmiL in C4-HSL synthesis. It showed that the production of violacein halo in 24 h culture was significantly increased in AmiL overexpressing strain, and decreased in deletion mutant (Fig. 6E). Taken together, these results indicate that AmiL directly targets PhzC, and negatively regulates *lasI* expression in early growth, but noticeably increases *rhlI* expression and C4-HSL production in the middle and late stages of growth.

## DISCUSSION

Here we demonstrate that the novel QS regulatory sRNA AmiL is involved in regulating a wide range of virulence phenotypes of P. aeruginosa, including pyocyanin, elastase, rhamnolipid, biofilm, swarming motility, hemolysin, and mammalian cytotoxicity. RNA-Seq result showed AmiL expression in PAO1 was negatively regulated by QS. Its transcription was probably repressed by RhlR. Further analysis on the mechanism of AmiL in regulating virulence, we found that AmiL directly targeted PhzC, and significantly regulated *lasI* and *rhlI* expression as well as C4-HSL production. Thus, our findings disclose a novel signaling cascade of *las*/*rhl* (RhlR)-AmiL-PhzC/*las*/*rhl*, and implicate the roles of AmiL in regulating PAO1 virulence.

As with most studies, we used RNA-Seq to characterize different QS regulatory sRNA species in P. aeruginosa. RNA-Seq technology has quickly become the new standard in transcriptome analysis of many bacterial studies (41, 42), and it also has been widely used to identify QS-controlled genes in P. aeruginosa (28–30). The data derived from our RNA-Seq experiments have allowed us to view the changes of PAO1 transcriptomes under QS activation or inhibition conditions in detail. During QS activation (*t* = 2 h, 4 h, 6 h), the expression of AmiL in RNA-Seq showed a significant decrease in the mid-exponential phase (*t* = 4 h) (Table S1, S2), while its expression did not change in qRT-PCR (Fig. 2). The inconsistency between RNA-Seq and qRT-PCR results may be related to the difference of detection sensitivity between these two techniques, and the rapid changes of growth status in the mid-exponential phase of PAO1 should also be considered. While consistently with RNA-Seq, AmiL expression (*t* = 6 h) detected by qRT-PCR in *lasI* or *rhlI* gene-deficient strain was significantly increased. These results suggest the involvement of *las* and *rhl* systems in negatively regulating AmiL transcription. In *las* and *rhl* systems, 3-oxo-C12-HSL and C4-HSL are important QS signal molecules produced by the autoinducer synthase LasI or RhlI, respectively. Upon binding with 3-oxo-C12-HSL and C4-HSL, the receptor proteins LasR and RhlR get activated and quickly activate the *las* and *rhl* systems. We thus investigated the influence of 3-oxo-C12-HSL and C4-HSL treatments on AmiL expression, with concentrations ranging from 5 to 40 μM according to reported studies that have demonstrated they can activate the *las* and *rhl* systems (25, 43–45). Our data showed that even a high concentration of 3-oxo-C12-HSL had little influence on AmiL expression, whereas C4-HSL treatment dramatically inhibited its expression in a dose-dependent manner (Fig. 2). It suggested that RhlR/C4-HSL network probably regulated AmiL expression. RhlR is also known as a transcription factor of the *rhl* system. We thus further investigated the transcription factor involved in this regulation and found that RhlR was probably the potential one that noticeably decreased AmiL expression (Fig. 2). However, other experiments that directly prove the role of RhlR, such as β-galactosidase assays which a DNA fragment of AmiL promoter carrying the predicted binding sites was cloned upstream of the β-galactosidase gene in the reporter plasmid, should also be considered.

It has been shown that the four known connected QS systems in P. aeruginosa are organized hierarchically. The *las* system is at the top of the signaling, which positively regulates the gene expression of RhlI, RhlR, and PqsR (14, 15). RhlR is also shown to upregulate the expression of *lasI* and consequently increase 3-oxo-C12-HSL production (46). In our study, the roles of AmiL in *las* system-related virulence phenotypes such as LasB elastases, LasA protease, and exotoxin A are different. The activity of elastase (*t* = 8 h) was inhibited by AmiL, while staphylolytic activity (*t* = 24 h) and exotoxin A activity (*t* = 24 h) did not (Fig. 3). These results are generally consistent with our consequent data that the expression of *lasI* was negatively regulated by AmiL in the early growth (*t* =4 h), but did not change in 12 h or 20 h culture (Fig. 6). This indicates that the regulatory role of AmiL in *las* system is mainly in the early growth of PAO1. However, the influence of AmiL on *rhl* system was positive in the middle and late stages of PAO1 growth. Of which, AmiL enhanced the synthesis of rhamnolipid (*t* = 16 h) (Fig. 3), and significantly increased *rhlI* expression (*t* = 12 h, 20 h) and C4-HSL production (*t* = 24 h) (Fig. 6). Therefore, the regulatory roles of AmiL in *las* and *rhl* systems are not contradictory in this study.

Pyocyanin is synthesized from chorismate by two *phzABCDEFG* operons, and by the *phzM* and *phzS* genes which catalyze the final steps in the synthesis (47). Our data from GFP reporter assays showed that the IntaRNA predicted target, PhzC, was a direct target of AmiL (Fig. 6). Certainly, the synthesis of pyocyanin is widely regulated by QS. Of which, PqsR, the transcriptional activator of *pqs* system, plays an important positive role (12, 48). Additional regulatory signals from the *las* and *rhl* systems are also involved. We thus investigated the influence of AmiL on *pqs* system, in addition to the *las* and *rhl* systems. However, the expression of PqsA synthase gene *pqsA* has no change in AmiL deletion mutant and overexpressing strains (data not shown). PqsA is an anthranilate-coenzyme A ligase, which activates anthranilate to initiate the first step of the biosynthesis of PQS signals (49). This suggests that the negative role of AmiL in pyocyanin production is mainly related to its direct target on PhzC, while *pqs* system upstream on the pyocyanin biosynthetic pathway is not involved.

Biofilm formation is a key example of P. aeruginosa community behavior which is regulated by QS. It has been reported that rhamnolipid plays an important role in safeguarding the architecture of biofilm (50). In addition, pyocyanin is also a vital component for biofilm maturation by promoting the release of extracellular DNA (51, 52). QS-mediated productions of rhamnolipid and pyocyanin are both positive regulators of biofilm formation. Given the results that AmiL oppositely regulated rhamnolipid and pyocyanin production (Fig. 3), this may explain the weak effect of AmiL’s role in biofilm formation.

Biofilm formation is also related to motility, and they often work inversely in P. aeruginosa (53, 54). It also has been reported that the synthesis of rhamnolipid is needed for swarming motility (55). To better understand the involvement of AmiL in biofilm formation and rhamnolipid production, we investigated the swarming and swimming motilities of both △AmiL and AmiL^+^ strains. As expected, AmiL promoted the swarming motility of PAO1 (Fig. 4). This indicates that AmiL’s roles in rhamnolipid production and swarming motility are positive. Rhamnolipid is an important virulence phenotype of the *rhl* system and strongly promotes swarming motility (56). Compared with the *las* system, our data from Fig. 6 suggest that AmiL may have a stronger influence on *rhl* system with an extended period of regulation. Thus, AmiL is on the one hand negatively regulated by RhlR in its transcription (Fig. 2), while it positively regulates the *rhl* system on the other hand (Fig. 6). This novel RhlR-AmiL-*rhl* signaling cascade of PAO1 can be reasonable. The QS of P. aeruginosa is a fine adjustment system that controls the expression of more than 300 genes involved in virulence regulation (6). There are both positive and negative feedback loops of the QS system. It is reported that the RhlR/C4-HSL network can activate its regulon and enhance the expression of *rhlI*, thus forming a positive feedback loop (57, 58). LasR also induces the expression of RsaL, a transcription repressor of *lasI*, which generates a negative feedback loop that counteracts part of the positive signal (59, 60). These positive and negative feedback loops balance the levels of signal molecules of QS. For *rhl*, under the control of both *las* and *pqs* systems, it functions as an executor to predominantly activated many QS-mediated virulence factors (61). To restrain the over-activation of QS signal, an appropriate negative feedback loop is necessary for the *rhl* system. Here we reveal that the *rhl* system negatively regulates AmiL transcription through RhlR, thus preventing AmiL from over-activating itself. Therefore, this RhlR-AmiL-*rhl* signaling cascade is beneficial to balance the virulence regulation of P. aeruginosa.

**FIG 7:**
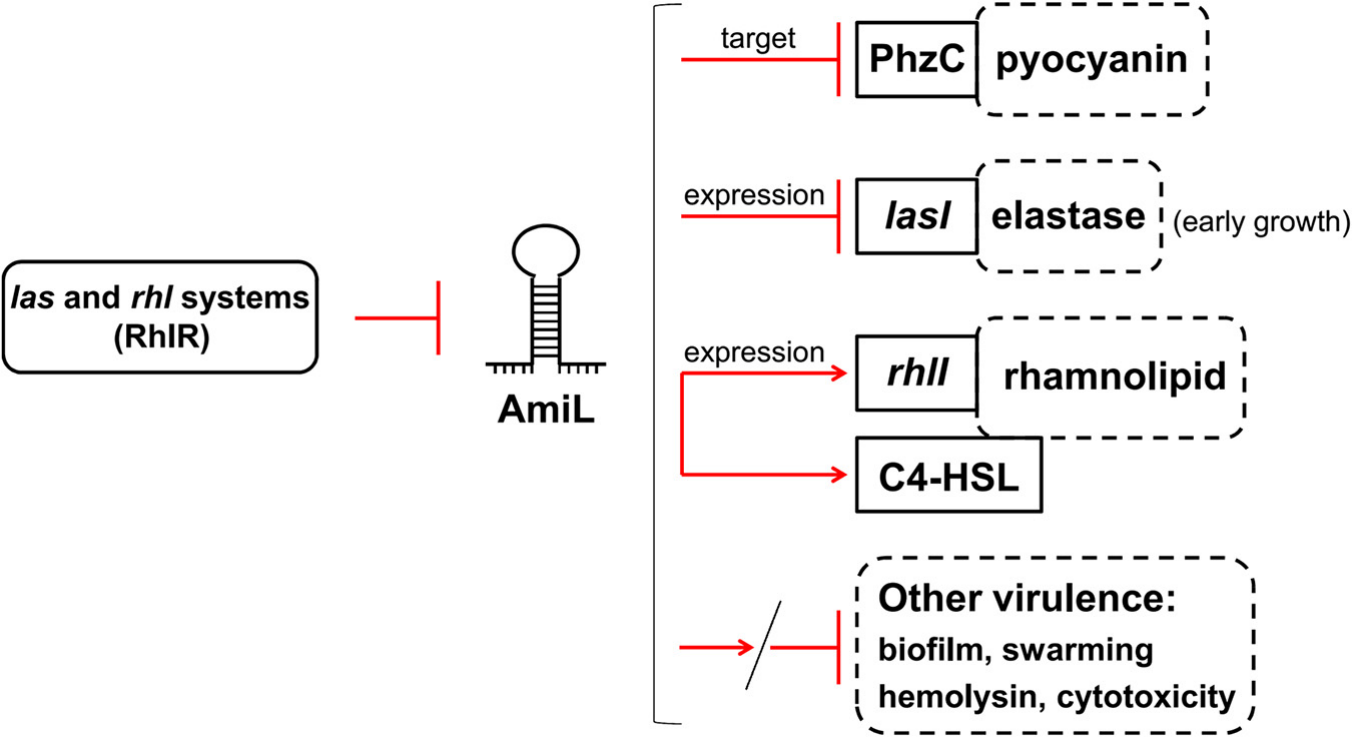
Schematic representation of a novel QS-sRNA regulatory network. The sRNA AmiL, negatively regulated by QS (probably RhlR), involves in regulating various QS-mediated virulence in PAO1, including pyocyanin, elastase, rhamnolipid, biofilm, swarming motility, hemolysin, and mammalian cytotoxicity. In addition to directly targeting PhzC, AmiL decreases *lasI* expression in the early growth of PAO1, but predominantly increased *rhlI* expression and C4-HSL production in the middle and late stages. Thus, this study clarified a novel signaling cascade of *las*/*rhl* (RhlR)-AmiL-PhzC/*las*/*rhl* in P. aeruginosa PAO1.

In summary, a novel QS-sRNA regulatory network of P. aeruginosa PAO1 is demonstrated in this study. Negatively regulated by QS (probably RhlR), AmiL involves in regulating various QS-mediated virulence phenotypes by targeting PhzC directly and modulating *las* and *rhl* systems (Fig. 7). Thus, our findings disclose an unstudied QS regulatory sRNA and advance the understanding of QS-sRNA signaling cascade in P. aeruginosa PAO1.

## MATERIALS AND METHODS

### Bacterial strains, plasmids, and growth conditions.

All bacterial strains and plasmids used in this study are listed in Table S5. PAO1 was a gift from Dr. Zhou Lin (Children’s Hospital of Chongqing Medical University). Unless indicated otherwise, bacteria cultures were grown at 37°C in LB medium or on LB plates containing 1.5% agar. When necessary, antibiotics were supplemented at the following concentrations: 100 μg/mL ampicillin (Amp), 30 μg/mL gentamicin (Gm), and 16 μg/mL chloramphenicol (Cm).

### Construction of the overexpression strains.

The EV pROp200, a Gm-resistant overexpression plasmid that can replicate stably in P. aeruginosa under the strong promoter P*tac*, was saved in our lab. This plasmid was used by Lu et al. in our previous study to construct *lasR or rhlR* gene-overexpressing strains, named LasR^+^ and RhlR^+^, respectively (25). These two strains were used in this study. Similarly, the whole sequence of *amiL* gene was amplified by PCR from PAO1 WT genomic DNA using the cloning primers AmiL-over-F and AmiL-over-R described in Table S6. PCR product and EcoRI-digested pROp200 plasmid were gel extracted, and then they were ligated using a Ready-to-Use Seamless Cloning Kit (BBI Life Science, Shanghai, China). The reaction solution was transformed into E. coli DH5α and selected with Gm. The constructed AmiL overexpressing plasmid (AmiL^+^) was confirmed by direct sequencing and then was transformed into PAO1 to generate the overexpression strain.

### Construction of the gene-deficient strains.

A sacB-based suicide vector system using the homologous recombinant plasmid pGSM was described by Zeng et al. in our previous study to construct the *lasI* or *rhlI* gene-deficient strain, named △*lasI* and △*rhlI* (62). These two strains were used in this study. Similarly, the upstream and downstream recombinant fragments of *amiL* were amplified by PCR using the cloning primers AmiL-P1, AmiL-P2, AmiL-P3, and AmiL-P4. The primers were listed in Table S6. The two PCR products were gel extracted, and then connected by fusion PCR to delete *amiL*. SacI and XbaI were used to digest pGSM plasmid and fusion PCR product, respectively. After digestion, they were ligated by T4 ligase and transformed into E. coli SM10λπ selected with Gm. Next, the gained SM10λπ pGSM-△AmiL strain was under conjugation with the PAO1 WT strain, selected by Amp and Gm. The clone that grew was the PAO1 pGSM-△AmiL strain, and it was reversely screened on a 10% sucrose-LB plate to generate the PAO1 △AmiL strain. Finally, the PAO1 △AmiL strain was transferred with pROp200 plasmid to construct the *amiL*-deleted PAO1 strain carrying pROp200 (△AmiL).

### RNA-Seq.

A single clone from the LB plate of strain PAO1, △*lasI*, and △*rhlI* were grown at 37°C 200 rpm in 4 mL LB medium overnight, then 500 μL culture was added to a new 4-mL LB medium for the second grow of 1 h, and 100 μL culture was added to another 4-mL fresh LB medium finally to incubate for the indicated time as described in Fig. 1B. Total RNA was extracted according to the manufacturer’s instructions using RNAprep Pure Cell/Bacteria Kit (TIANGEN Biotech, Beijing, China), from three biological replicates of either strain as described above. The entire transcriptome sequencing (RNA-Seq) and subsequent bioinformatics analysis were conducted by Science Corporation of Gene Co., Ltd (Guangzhou, China).

### qRT-PCR.

A single clone from the LB plate of indicated strain was grown at 37°C 200 rpm in 3 mL LB medium overnight, then 30-μL culture was added to a new 3-mL LB medium (cell density equivalent) for the second grow for the indicated time. After collecting the culture, total RNA was extracted using TRIzol Reagent (Invitrogen, Carlsbad, CA) and quantified on a NanoDrop 2000 spectrophotometer. Reverse transcription was then performed with PrimeScript RT reagent kit (TaKaRa, Dalian, China), in which 1 μg RNA was under reverse transcription in a total reaction volume of 20 μL. Next, quantitative PCR was performed in triplicate with each cDNA template using SYBR green Premix Pro *Taq* HS qPCR Kit (Accurate Biology, Changsha, China), and performed on a ViiA^TM^ 7 Dx system (Applied Biosystems, Foster, CA). Quantification cycle values were normalized to the housekeeping gene rpoD using the relative threshold cycle (2^−ΔΔCt^) method. qRT-PCR primers used are described in Table S6.

### QS signal molecules treatment.

A single clone from the LB plate of PAO1was grown at 37°C 200 rpm in 3-mL LB medium overnight, then 30-μL culture was added to a new 3-mL LB medium for the second grow for 6 h with different concentrations of 3-oxo-C12-HSL (Sigma-Aldrich, St. Louis, MO) or C4-HSL (Sigma-Aldrich, St. Louis, MO). The used concentrations ranged from 5 to 40 μM according to reported studies (25, 43–45), and results compared with the vehicle DMSO (0 μM). The expression levels of AmiL were measured by qRT-PCR as described above.

### Pyocyanin production assays.

The indicated strain was grown in LB medium overnight, then 60-μL culture was added to a new 6-mL LB medium to have a second grow at 37°C 200 rpm for 24 h. The culture supernatant was then collected by centrifugation, and pyocyanin was extracted with appropriate chloroform and 0.2N HCl. The absorbances of the extraction solutions were measured at 520 nm and 600 nm in a BioTek Synergy H1 microplate reader (BioTek, Winooski, VT). The concentration of pyocyanin was calculated using the following formula: (A520/A600 × 17.072) = μg/mL (63). Relative pyocyanin level was described according to the determined concentration value.

### Elastase assays.

Elastase activity of P. aeruginosa was determined by elastin-Congo red (ECR) assays (64). Then 20 μL of the indicated strain under mid-exponential phase were inoculated into 2-mL LB medium at 37°C 200 rpm for 8 h. Then 200-μL culture supernatant was added to 800 μL ECR (Sigma-Aldrich, St. Louis, MO) solution which was containing 0.1 M Tris (pH 7.2), 1 mM CaCl_2_, and 3 mg/mL ECR at 37°C 200 rpm for 4 h. Finally, 100 μL of 0.12 M EDTA was added to terminate the enzymatic reaction. Insoluble ECR was removed by centrifugation, and 200 μL of supernatant was taken to measure the OD495.

### Biofilm formation assays.

The indicated strain was grown in LB medium overnight, and the culture solution was then mixed with fresh LB medium at a ratio of 1:100. Next, 100 μL of the mixed solution was added to a 96-well plate and incubated at 37°C for 24 h. After gently washing three times with 0.9% NaCl to remove planktonic cells, the biofilm cells attached to the plate were fixed with methanol for 5 min and then stained with 1% crystal violet. Finally, 95% ethanol was used to solubilize the stained biofilm cells, and 200 μL of supernatant was taken to measure the OD600.

### Rhamnolipid assays.

The production of rhamnolipid was measured by methylene blue complexation (65). Then 20 μL of the indicated strain under mid-exponential phase was inoculated into 2 mL M9 medium (containing 0.4% glucose, 2 mM MgSO4, and 100 μM CaCl_2_) and grown at 37°C 200 rpm for 16 h. Also, 1 mL of culture supernatant was acidified with 40 μL 1 N HCl, and rhamnolipid was extracted with 4 mL chloroform. Finally, 3 mL of chloroform extract was reacted with 100 μL of methylene blue (1 g/l) and appropriate distilled water, and 200 μL of chloroform layer was taken to measure the OD638.

### LasA staphylolytic assays.

30 μL of the indicated PAO1 strain under the mid-exponential phase was inoculated into 3 mL fresh LB medium and grown at 37°C 200 rpm for 24 h. Meanwhile, Staphylococcus aureus (S. aureus) was cultured in LB medium at 37°C overnight and subsequently washed and resuspended with 0.9% NaCl. The S. aureus saline solution was boiled at 105°C for 15 min and the cell pellet was collected and resuspended in 0.9% NaCl (OD600 ≈ 0.8). Next, 100 μL of PAO1culture supernatant was added with 900 μL of the treated S. aureus solution, and the OD600^a^ was measured. After the culture at 37°C for 6 h, 200 μL of supernatant was again taken to measure the OD600^b^. S. aureus solution treated with LB medium was used as a negative control. S. aureus lysis (%) was calculated using the following formula: (OD600^a^ − OD600^b^)/OD600^a^ × 100%.

### Exotoxin A assays.

Thirty μL of the indicated strain under mid-exponential phase were inoculated into 3-mL Trypticase soy broth (TSB) medium with 1% glycerol and grown at 37°C 200 rpm for 24 h; 100 μL of culture supernatant was added to 100 μL NAD (0.25 mg/mL) solution and then was mixed and water bathed at 25°C for 30 min. Next, color-substrate solution containing 0.2 mL INT (4 mg/mL), 0.8 mL PMS (1 mg/mL), 0.1 mL LDH (5 mg/mL), and 0.1 mL sodium lactate (0.5 mol/L) was added, and phosphate-buffered saline (PBS) buffer (10 mmol/L, pH 7.4) was also added to the reaction solution. After water bathing at 37°C for 5 min, 0.1 mL HCl (2 mol/L) was used to terminate the reaction, the OD490 was measured.

### Motility assays.

Five μL of the indicated strain under mid-exponential phase was spotted onto the middle of swarming medium (LB containing 0.5% agar and 5 g/L glucose) and swimming medium (tryptone broth containing 0.3% agar). The results of motility assays were observed after 16 h of incubation at 37°C.

### Human blood hemolysis assays.

Whole blood from 2 to 3 clinically healthy individuals was collected. The leukocytes layer was removed by centrifugation at 2,000 rpm for 5 min and was washed and resuspended with PBS buffer (pH 7.4) to dilute to 4% washed erythrocytes. Next, 300 μL of the indicated strain under mid-exponential phase was also washed and resuspended with PBS buffer, and then was added to 300 μL erythrocytes suspension in a sterile EP tube (2% Triton X-100 was used as a positive control, PBS as a negative control). The coculture tubes were incubated at 37°C 200 rpm for 4 h, and the supernatant after centrifugation was finally added to a 96-well plate to measure the absorbance at 450 nm. The hemolysis rate (%) was calculated using the following formula: (A_coculture_ − A_PBS_)/(A_Triton X-100_ − A_PBS_) × 100%.

### Cytotoxicity assays.

Human pulmonary epithelial cell line A549 (ATCC: CCL-185) cells were cultured in Dulbecco's modified Eagle medium (DMEM) (Gibco, Carlsbad, CA) complemented with 10% FBS (Gibco, Carlsbad, CA) at 37°C with 5% CO_2_, and seeded on a 96-well plate (3 × 10^4^/well) 1 day before infection. Next, the indicated bacteria strains under mid-exponential phase were collected. The medium of A549 cells was removed and replaced with a suspension containing 6 × 10^5^ CFU/mL of bacteria (MOI of 4, 200 μL/well). The coculture was incubated at 37°C with 5% CO_2_ for 12 h, followed by monitoring the release of lactate dehydrogenase (LDH) as an indicator of cytotoxicity according to the manufacturer’s instructions using the LDH Cytotoxicity Detection Kit (Beyotime, Shanghai, China). LDH release buffer from the kit was used as a positive control. The absorbances at 490 nm and 900 nm were read, and the absorbance at 900 nm was subtracted from the absorbance at 490 nm. Percent cytotoxicity was calculated using the following formula: (A_coculture_ − A_cell control_)/(A_positive control_ − A_cell control_) × 100%.

### Fluorescent reporter assays.

A GFP reporter plasmid (pUCP30T-*gfp*) from our lab was used (25). The sequence of WT PhzC mRNA containing putative binding sites for AmiL was amplified by PCR from PAO1WT genomic DNA, using the cloning primers described in Table S6. Then the sequence was inserted into the XbaI/NcoI sites upstream of the first codon of GFP in pUCP30T-*gfp* plasmid to gain a pUCP30T-*phzC*-*gfp* plasmid, confirmed by direct sequencing. Next, this constructed plasmid was co-transformed with pSTV28-*amiL* (AmiL^+^*) or with pSTV28 (EV*) into E. coli DH5α. For mutant (Mut) plasmid, pUCP30T-mut-*phzC*-*gfp*, which carried a mutated sequence in the complementary sites for AmiL was generated using fusion PCR. The sequence chosen for mutation was CGGTCCCGCAGATC, and was mutated to CAAAGGGCGTCATC. After co-transformation, the cultured DH5α strains were collected by centrifugation, and washed and resuspended in 0.9% NaCl. Finally, 200 μL of the strain solution was transferred to a black polystyrene 96-well plate. The fluorescence intensity (F485/535) was measured in a BioTek Synergy H1 microplate reader (BioTek, Winooski, VT). GFP activity was expressed in arbitrary units (AU) as F485/535. Then 10 μL of the strain solution was also added to a slide to observe the fluorescence by Nikon ECLIPSE Ti2-U fluorescence microscope (Nikon, Tokyo, Japan).

### AHL reporter plate bioassay.

Chromobacterium violaceum strain CV026 was used as a biosensor to visualize C4-HSL production by P. aeruginosa. This violacein production in CV026 is inducible by AHLs with N-acyl side chains from C4 to C8 in length (38–40). The CV026 cultures under mid-exponential phase were added to a warm LB agar (1.5%) medium at a ratio of 1:20, and then the CV026-LB mixtures were poured immediately over the surface of LB agar plates prepared in Petri dishes. When the overlaid agar had solidified, a well (5 mm diameter) was made in the center of each plate. Next, 40 μL of the indicated PAO1 strain under mid-exponential phase was added to the well. Violacein halo production was observed after incubation at 30°C for 24 h. The diameter of the violacein halo was also measured.

### Statistical analysis.

For each separate set of assays, at least three independent experiments were performed. Data are shown as mean ± SEM by using GraphPad Prism 5 (GraphPad Software, San Diego, CA). Statistical significance was determined by Student’s *t* test between two groups, with *P* values represented as *, *P < *0.05; **, *P < *0.01; and ***, *P < *0.001.

### Data availability.

All the RNA-Seq data in this study was available through the public database NCBI BioProject (Accession No. PRJNA795818).
