# Supplementary material for: The Small RNA AmiL Regulates Quorum Sensing-Mediated Virulence in Pseudomonas aeruginosa PAO1
Source: Microbiol Spectr. 2022 Mar 9;10(2):e02211-21. doi: 10.1128/spectrum.02211-21 (PMC9045362; doi:10.1128/spectrum.02211-21)
Supplement: SUPPLEMENTAL FILE 1 — Supplemental material. Download spectrum02211-21_supp_1_seq11.pdf, PDF file, 0.2 MB [file spectrum02211-21_supp_1_seq11.pdf]

TABLE S1 Selected sRNAs with differential CPM in QS activation and QS gene-deficient conditions by RNA-Seq

Locus tag	Name	CPM (counts per million)				
		PAO1 *2 h*	PAO1 *4 h*	PAO1 *6 h*	△*lasI 6 h*	△*rhlI 6 h*
PA0836.1	P5	125.74	99.49	267.86	244.91	311.17
PA1112.1		41.51	59.06	70.23	83.99	87.16
PA0887.1	P7	24.39	51.67	52.60	52.85	49.33
PA2942.1	P15	23.83	14.15	10.82	19.79	20.64
PA2958.1	RgsA	147.93	240.68	190.79	190.04	163.95
PA3304.1	P18	21.08	21.04	33.07	25.73	29.27
**PA3366.1**	**AmiL**	**46.32**	**27.33**	**44.02**	**149.76**	**103.07**
PA3621.1	RsmZ	24.59	18.84	19.23	22.07	22.72
PA4406.1		856.69	1150.08	934.80	1050.25	1075.37
PA4421.1	RnpB	87324.48	148798.32	194601.64	221663.35	185747.71
PA4451.1	P35	32.43	25.31	18.41	20.68	24.55
PA4704.1	PrrF1	38.36	78.48	73.60	78.02	79.74
PA4704.2	PrrF2	7.60	11.59	11.53	14.98	12.22
PA4758.1	P32	66.47	58.64	83.67	62.43	70.44
PA5227.1	SsrS	446.48	347.28	603.42	758.26	870.71

The calculation of Unigene expression uses a normalization method, CPM, counts of exon model per million mapped reads.

TABLE S2 Selected sRNAs with differential expression in QS activation condition by RNA-Seq

Locus tag	Name	PAO1 *2 h* vs. *4 h*		PAO1 *2 h* vs. *6 h*		PAO1 *4 h* vs. *6 h*	
		logFC	*P*	logFC	*P*	logFC	*P*
PA0836.1	P5	0.60	6.06E-03	-0.76	9.62E-06	-1.38	5.55E-09
PA1112.1		-0.24	9.93E-02	-0.43	7.16E-04	-0.21	1.23E-01
PA0887.1	P7	-0.81	6.48E-03	-0.78	3.19E-06	0.01	9.75E-01
PA2942.1	P15	0.99	9.07E-04	1.45	5.76E-08	0.44	3.49E-02
PA2958.1	RgsA	-0.43	3.84E-02	-0.03	7.92E-01	0.37	5.29E-02
PA3304.1	P18	0.27	2.54E-01	-0.33	8.29E-02	-0.62	5.37E-04
**PA3366.1**	**AmiL**	**1.02**	**2.79E-05*****	**0.41**	**1.47E-01**	**-0.64**	**1.58E-02***
PA3621.1	RsmZ	0.65	8.21E-03	0.67	2.40E-03	0.00	1.00E+00
PA4406.1		-0.15	3.64E-01	0.21	1.56E-01	0.34	4.53E-02
PA4421.1	RnpB	-0.50	1.55E-02	-0.82	6.70E-05	-0.34	1.38E-01
PA4451.1	P35	0.62	5.49E-03	1.14	8.42E-08	0.50	2.25E-02
PA4704.1	PrrF1	-0.74	1.97E-02	-0.59	4.00E-03	0.12	6.36E-01
PA4704.2	PrrF2	-0.35	1.25E-01	-0.27	1.98E-01	0.05	7.98E-01
PA4758.1	P32	0.46	1.55E-02	0.00	1.00E+00	-0.48	9.95E-03
PA5227.1	SsrS	0.63	7.43E-04	-0.11	6.92E-01	-0.76	1.27E-02

logFC, $\log_2$ (Fold Change); *P* value, *, $P < 0.05$; ***, $P < 0.001$.

TABLE S3 Selected sRNAs with differential expression in QS gene-deficient condition by RNA-Seq

Locus tag	Name	PAO1 *6 h* vs. △*lasI 6 h*		PAO1 *6 h* vs. △*rhlI 6 h*	
		logFC	*P*	logFC	*P*
PA0836.1	P5	0.10	6.65E-01	-0.20	3.98E-01
PA1112.1		-0.28	5.95E-02	-0.29	7.79E-02
PA0887.1	P7	-0.02	9.00E-01	0.11	4.97E-01
PA2942.1	P15	-0.91	2.36E-02	-0.92	4.10E-02
PA2958.1	RgsA	-0.02	9.19E-01	0.24	9.46E-02
PA3304.1	P18	0.35	6.10E-02	0.20	2.97E-01
**PA3366.1**	**AmiL**	**-1.80**	**1.69E-04*****	**-1.22**	**2.67E-02***
PA3621.1	RsmZ	-0.23	3.83E-01	-0.21	3.47E-01
PA4406.1		-0.19	2.15E-01	-0.18	3.24E-01
PA4421.1	RnpB	-0.22	3.02E-01	0.09	5.65E-01
PA4451.1	P35	-0.22	4.21E-01	-0.38	1.65E-01
PA4704.1	PrrF1	-0.11	5.12E-01	-0.09	4.95E-01
PA4704.2	PrrF2	-0.39	1.22E-01	-0.07	7.66E-01
PA4758.1	P32	0.41	1.55E-02	0.27	1.02E-01
PA5227.1	SsrS	-0.35	2.19E-01	-0.51	9.92E-02

logFC, $\log_2$ (Fold Change); *P* value, *, $P < 0.05$; ***, $P < 0.001$.

TABLE S4 The *amiEBCRS* operon genes with differential CPM in QS activation and QS gene-deficient conditions by RNA-Seq

Locus tag	Name	CPM (counts per million)				
		PAO1 *2 h*	PAO1 *4 h*	PAO1 *6 h*	△*lasI 6 h*	△*rhlI 6 h*
PA3366	amiE	62.99	92.35	202.86	572.41	367.28
PA4947	amiB	193.54	227.36	229.25	245.98	257.83
PA3364	amiC	13.69	43.24	118.04	323.92	182.47
PA3363	amiR	13.84	41.00	122.15	336.01	188.34
PA3362	amiS	9.37	16.11	67.50	95.99	67.14

The calculation of Unigene expression uses a normalization method, CPM, counts of exon model per million mapped reads.

TABLE S5 Bacterial strains and plasmids

Strains / Plasmids	Characteristics	Source
Pseudomonas		
PAO1	Wild-type strain, Gm^S, Amp^R	Our lab
PAO1 △*lasI*	Deficiency of *lasI*, Gm^S, Amp^R	Our lab (1)
PAO1 △*rhlI*	Deficiency of *rhlI*, Gm^S, Amp^R	Our lab (1)
PAO1 △AmiL	Deficiency of *amiL*, Gm^S, Amp^R	This work
Escherichia coli		
DH5α	Wild-type strain, Gm^S, Amp^S	Our lab
SM10λπ	Wild-type strain, Gm^S, Amp^S	Our lab
Plasmids		
pGSM	Homologous recombinant plasmid, Gm^R, Amp^R	Our lab (1)
pROp200 (EV)	Control plasmid based on pBBR1 MCS-5, Gm^R	Prof. Shiyun Chen (2)
pROp200-LasR$^+$	PAO1 *lasR* overexpression plasmid, controlled by P_{tac} promoter, Gm^R	Our lab (3)
pROp200-RhlR$^+$	PAO1 *rhlR* overexpression plasmid, controlled by P_{tac} promoter, Gm^R	Our lab (3)
pROp200-AmiL$^+$	PAO1 *amiL* overexpression plasmid, controlled by P_{tac} promoter， Gm^R	This work
pSTV28 (EV*)	Control plasmid, containing P_{lac} promoter, Cm^R	Our lab
pSTV28-AmiL^{+*}	PAO1 *amiL* overexpression plasmid, controlled by P_{lac} promoter, Cm^R	This work
pUCP30T-*gfp*	Control plasmid, *gfp* controlled by P_{lac} promoter, Gm^R	Our lab(3)
pUCP30T-*lasI-gfp*	Containing transcription fusion of *lasI-gfp*, which controlled by P_{lac} promoter, Gm^R	This work
pUCP30T-*rhlR-gfp*	Containing transcription fusion of *rhlR-gfp*, which controlled by P_{lac} promoter, Gm^R	This work
pUCP30T-*phzC-gfp*	Containing transcription fusion of *phzC-gfp*, which controlled by P_{lac} promoter, Gm^R	This work
pUCP30T-mut-*phzC-gfp*	Containing transcription fusion of mut-*phzC-gfp*, which controlled by P_{lac} promoter, Gm^R	This work

Gm, Amp and Cm stand for gentamicin, ampicillin and chloramphenicol, respectively. S, sensitive; R, resistant.

TABLE S6 Sequences of cloning primers and qRT-PCR primers

**Name**	**Purpose**	**Sequence (5'→3')**
Cloning primers		
AmiL-P1	△AmiL	ACGGCCAGTGAATTCGAGCTCAAAGCGCCAGTTTGTAGCAG
AmiL-P2	△AmiL	CATGGATATCACCTCCTTATAAGCCCCGTCGGAAG
AmiL-P3	△AmiL	GACGGGGCTTATAAGGAGGTGATATCCATGCGTCA
AmiL-P4	△AmiL	GGCTGGATCCCAAGCTCTAGATCCTTCTCCAGTCCCTCGTA
AmiL-over-F	$AmiL^{+}$	ATCGGCTCGTATAATGAATTCATCAGGTCGTGCGCATCA
AmiL-over-R	$AmiL^{+}$	CGAATTTTAACAAAAGAATTCGGATATCACCTCTTGTTGTT
In28-AmiL-F	$AmiL^{+*}$	TCCTCTAGAGTCGACCTGCAGCTTGCCCGAGCCTTGACAGC
In28-AmiL-R	$AmiL^{+*}$	ACGACGGCCAGTGCCAAGCTTTTGACCACCGCCACTCCGA
30T-*lasI*-F	*lasI-gfp*	GCTCTAGAATGAGCCGTTTCGCCATCA
30T-*lasI*-R	*lasI-gfp*	CATGCCATGGTGCCGATCTTCAGGTGC
30T-*rhlR*-F	*rhlR-gfp*	CGGAATTCATGCACGATTCCCTTCACC
30T-*rhlR*-R	*rhlR-gfp*	GCTCTAGAATGCTCCAGGTCGGTCA
30T-*phzC*-F	*phzC-gfp*	GCTCTAGAATGCCACTTCCTGCATTCCTTC
30T-*phzC*-R	*phzC-gfp*	CATGCCATGGGATCGTCCATAGTTCTCCC
30T-mut-*phzC*-F	mut-*phzC-gfp*	ATTCCAAAGGGCGTCATCAAACGCGAAGGC
30T-mut-*phzC*-R	mut-*phzC-gfp*	TTGATGACGCCCTTTGGAATGCTCAGGGCG
qRT-PCR primers		
rpoD-F	qRT-PCR	CTGAAGATCGCCAAAGAGCC
rpoD-R	qRT-PCR	GTGTGGTCGGTGTTCATGTC
amiL-F	qRT-PCR	GCATCAGCGTCGATGT
amiL-R	qRT-PCR	AGCCCATTTGCTCTGT
lasI-F	qRT-PCR	CGTGCTCAAGTGTTCAAGGA
lasI-R	qRT-PCR	AAAACCTGGGCTTCAGGAGT
rhlI-F	qRT-PCR	CTACCGGCATCAGGTCTTCA
rhlI-R	qRT-PCR	GTTTCGCTGCACAGGTAGG

References:

1. Zeng J, Zhang N, Huang B, Cai R, Wu B, E S, Fang C, Chen C. 2016. Mechanism of azithromycin inhibition of HSL synthesis in *Pseudomonas aeruginosa*. Sci Rep 6:24299. https://doi: 10.1038/srep24299.
2. Lu P, Wang Y, Zhang Y, Hu Y, Thompson KM, Chen S. 2016. RpoS-dependent sRNA RgsA regulates Fis and AcpP in Pseudomonas aeruginosa. Mol Microbiol 102:244-259. https://doi: 10.1111/mmi.13458.
3. Lu Y, Li H, Pu J, Xiao Q, Zhao C, Cai Y, Liu Y, Wang L, Li Y, Huang B, Zeng J, Chen C. 2019. Identification of a novel RhlI/R-PrrH-LasI/Phzc/PhzD signalling cascade and its implication in *P. aeruginosa* virulence. Emerg Microbes Infect 8:1658-1667. https://doi: 10.1080/22221751.2019.1687262.
